# Synthesis of Bisphenol A Based Phosphazene-Containing Epoxy Resin with Reduced Viscosity

**DOI:** 10.3390/polym11121914

**Published:** 2019-11-20

**Authors:** Vyacheslav V. Kireev, Yulya V. Bilichenko, Roman S. Borisov, Jianxin Mu, Dmitry A. Kuznetsov, Anastasiya V. Eroshenko, Sergey N. Filatov, Igor S. Sirotin

**Affiliations:** 1 Department of Plastics, Mendeleev University of Chemical Technology of Russia, Miusskaya sq. 9, 125047 Moscow, Russia; kireev@muctr.ru (V.V.K.); julyab2@gmail.com (Y.V.B.); eroshenko.nast@yandex.ru (A.V.E.); filatovsn@muctr.ru (S.N.F.); 2 Topchiev Institute of Petrochemical Synthesis, Russian Academy of Sciences, Leninskii pr. 29, 119991 Moscow, Russia; borisov@ips.ac.ru; 3 Department of Organic Chemistry, Peoples′ Friendship University of Russia, Miklukho-Maklaya str.6, 117198 Moscow, Russia; 4 College of Chemistry, Jilin University, 2699 Qianjin Street, Changchun 130012, China; emujianxin@163.com; 5 Scientific & Research Institute of Natural Gases and Gas Technologies—Gazprom VNIIGAZ, Razvilka, s.p. Razvilkovskoe, Leninsky dist., Moscow region, 142717 Moscow, Russia; expdk@yandex.ru

**Keywords:** epoxy resin, epoxy oligomer, phosphazene, bisphenol A, phenol

## Abstract

Phosphazene-containing epoxy oligomers (PEO) were synthesized by the interaction of hexachlorocyclotriphosphazene (HCP), phenol, and bisphenol A in a medium of excess of epichlorohydrin using potassium carbonate and hydroxide as HCl acceptors with the aim of obtaining a product with lower viscosity and higher phosphazene content. PEOs are mixtures of epoxycyclophosphazene (ECP) and a conventional organic epoxy resin based on bisphenol A in an amount controlled by the ratio of the initial mono- and diphenol. According to ^31^P NMR spectroscopy, pentasubstituted aryloxycyclotrophosphazene compounds predominate in the ECP composition. The relative content in the ECP radicals of mono- and diphenol was determined by the MALDI-TOF mass spectrometry method. The organic epoxy fraction, according to gas chromatograpy-mass spectrometry (GC-MS), contains 50–70 wt % diglycidyl ether of bisphenol A. PEO resins obtained in the present work have reduced viscosity when compared to other known phosphazene-containging epoxy resins while phosphazene content is still about 50 wt %. Resins with an epoxy number within 12–17 wt %, are cured by conventional curing agents to form compositions with flame-retardant properties, while other characteristics of these compositions are at the level of conventional epoxy materials.

## 1. Introduction

Out of all the varieties of synthetic polymers, epoxies, developed over 70 years ago, won a special place in industry and everyday life, not necessarily in terms of overall manufacturing volume, moreso in their specific role [1]. Classic bisphenol A based epoxy resins have many advantages: low cost, low curing shrinkage, good chemical resistance, and mechanical properties [1,2]. In terms of valuable qualities, epoxy polymers are superior to many other classes of synthetic polymers, which makes them indispensable as a basis for adhesives, paints, coatings, and binders for reinforced plastics [1,3]. The development and application of new epoxy oligomers and binders based on them are expanding at a rapid pace despite the appearance of new generation binders, such as bismaleimides, polyimides, and cyanate esters [2]. The latter are characterized by enhanced physical and mechanical properties, including high heat resistance and low flammability. However, their price is very high, their processing properties are worse than those of the same epoxides, and a high degree of crosslinking can adversely affect the mechanical properties [1,2].

However, polymers based on unmodified epoxy resins often have low and unstable performance characteristics, in particular flammability and relatively low heat resistance [3]. 

A rather effective way to increase the heat resistance of epoxy matrices is with structural modification, for example, with compatible oligomers of higher functionality, which are incorporated into the three-dimensional network structure formed during curing. Examples of such resins are triglycidyl-p-aminophenol (TGPAP), tetraglycidyl-4,4′-methylenedianiline (TGMDA), and epoxidized novolacs [3,4].

The solution of the flammability problems of epoxy polymers is a more complex issue and involves trade-offs. Thus, a traditional and effective way to reduce the flammability of epoxy systems is to replace bisphenol A based epoxy resins with their brominated analogs. However, brominated epoxy resins, as well as other halogen-containing compounds, emit toxic gases when they come into contact with flames, which limits their use, for example, in civil aviation and transport, for environmental reasons [5,6]. 

One of the promising ways of modifying epoxy polymers to increase heat and fire resistance is the introduction of organometallic compounds, in particular phosphorus compounds, known as universal flame retardants for a large number of polymeric materials. Refusal of halogen-containing flame retardants in favor of phosphorus-based ones is a modern global trend [5].

However, the introduction of low-molecular, non-reactive compounds into the material, including red phosphorus and its inorganic compounds (phosphates, polyphosphates, etc.) worsens the mechanical properties and transparency of polymers [5]. Organic phosphates, which are better combined with the base polymer, have proven themselves more functional. Meanwhile, there are some difficulties associated, for example, with the fact that ethers of phosphoric acids can act as plasticizers, reducing the heat resistance of the material [7]. The best properties are possessed by binders containing functional organic phosphorus compounds capable of forming covalent bonds with an epoxy matrix. [6,8]. For example, glycidyl ethers of phosphorus acids, which not only reduce the flammability, but also increase the mechanical and adhesive strength of the material, are quite promising structural phosphorus-containing modifiers [8,9]. However, they are also poorly compatible with epoxy polymers [9,10]. Finally, most industrial phosphorus-containing flame retardants have a much lower degradation temperature than epoxy polymers [5,6,11,12], which makes it impossible to use them in engineering plastics and as part of high-temperature binders.

The combination of the above factors leads to the fact that epoxy resins are not always able to satisfy the growing needs of high-tech industries, especially in the aerospace industry, automotive industry, electrical engineering, electronics, etc.

A possible way to solve the problems described above, including the flammability of epoxy resins, is the use of modifiers based on phosphazenes.

The main chain of organophosphazenes consists of alternating atoms of phosphorus and nitrogen, and at the phosphorus atom there are organic radicals introduced by the substitution of halogen in halogenphosphazenes. The nature of organic substituents, usually introduced by the reaction of nucleophilic substitution of chlorine, can vary widely and determines the properties of the final polymer or oligomer. The unique properties of various organophosphazenes cause the ever-growing interest of researchers in phosphazene chemistry [13]. Compared to other organophosphorus compounds, aryloxyphosphazenes have, as a rule, higher thermal stability and chemical resistance and are promising non-halogen flame-retardants [6,14,15], characterized by the synergistic action of phosphorus and nitrogen [14,16]. Thus, phenoxycyclophosphazenes were commercialized as a flame retardant by Otsuka Chemical and others [14,17].

Researchers have long been trying to combine the exceptional properties of organophosphazenes as highly effective flame retardants with the function of structural modifiers, such as polyfunctional epoxy resins. There are two main synthetic approaches that allow to obtain functional phosphazenes capable of forming covalent bonds with epoxy matrices:

(1) The synthesis of organophosphazenes with reactive epoxy groups for addition to the epoxy component [18,19,20,21,22,23,24,25,26,27,28,29,30,31,32,33,34,35,36,37,38,39,40,41,42,43];
(2) The synthesis of organophosphazenes with reactive amine groups for use as a curing agent or its component [44,45,46,47,48,49,50,51,52].

Functional epoxyphosphazenes are highly effective flame retardants that not only do not reduce mechanical properties, since they are well compatible with the epoxy matrix, but can also improve them, probably due to the formation of a special three-dimensional polymer network, in the nodes of which phosphazene cycles are located [53].

Currently, the majority of methods for the synthesis of functional epoxyphosphazenes described in the literature are of primarily scientific interest due to the complexity of scaling and the large number of intermediate stages [18,19,20,21,22,24,28,30,31,33,34,35,36,38,39,40,41,42,43]. Although there are epoxyphosphazenes that are fairly easy to synthesize, for example, on the basis of hexachlorocyclo triphosphazene and glycidol [23,25,26,27,29,32,37,40]. However, alkoxyphosphazenes, which include such glycidyloxyphosphazenes, are not thermally stable [13]. Thus, even during their synthesis, an undesirable phosphazene-phosphazane rearrangement occurs [27]. Thus, only aromatic organophosphazenes can be used as a component of high-temperature epoxy binders.

**Figure 1:**
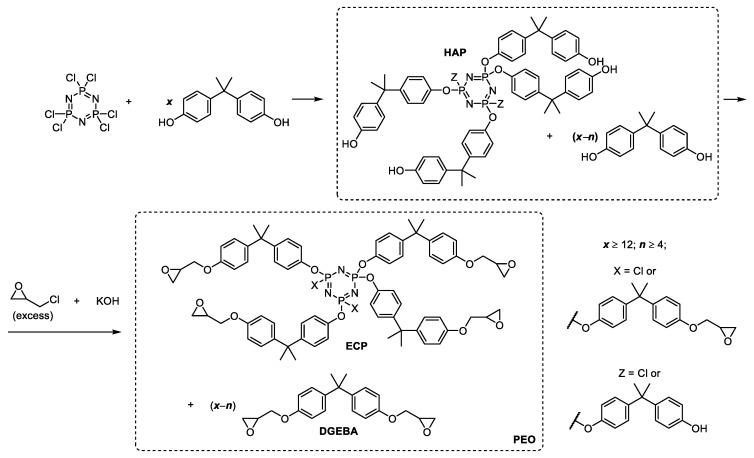
Synthesis of phosphazene-containing epoxy oligomers by the reaction of epichlorohydrin with hydroxyaryloxycyclotriphosphazenes, the condensation products of hexachlorocyclotriphosphazene and 4,4′-dioxydiphenyl-2,2-propane [55]. Hydroxyaryloxycyclotriphosphazenes (HAP), epoxycyclophosphazene (ECP), diglycidyl ether of bisphenol A (DGEBA), phosphazene-containing epoxy oligomers (PEO).

In recent years, phosphazene-containing epoxy oligomers (PEO) with reduced flammability on the base of cyclic chlorophosphazenes have been synthesized and characterized [54]. The most accessible and promising are PEOs obtained by the reaction of epichlorohydrin with hydroxyaryloxycyclotriphosphazenes (HAP), the condensation products of hexachlorocyclotriphosphazene (HCP) and 4,4′-dioxydiphenyl-2,2-propane (Figure 1).

The main problem in the synthesis of PEO is the high functionality of HCP, which requires the use of a more than 10-fold molar excess of diphenol to achieve a more complete replacement of the chlorine atoms in it and to avoid gelation. As for the industrial use of epoxyphosphazenes, the main limiting factor complicating the preparation of the formulation and worsening its processing properties is that most epoxyphosphazenes are solids with a softening temperature of 80–100 ° C and high melt viscosity. High average functionality can also influence processing properties.

Epoxidation of a mixture of HAP and excess of diphenol with epichlorohydrin produces PEO, which contains fractions of the usual bisphenol A based epoxy resin and epoxycyclophosphazene (ECP) oligomers with a variable ratio [54]. In [55,56], single-stage synthesis of PEO was realized by direct interaction of HCP and an excess of diphenol (bisphenol A [55,56] or resorcinol [57]) in the epichlorohydrin medium as a reagent and a solvent. The resulting PEO includes ECP consisting essentially of tetra- and pentaepoxides of the above formula with *n* = 4 and 5 and a conventional organic epoxy monomer. These PEOs in comparison with pure epoxyphosphаzenes contain organic epoxide, which is in fact an active diluent that lowers the viscosity and average functionality to an acceptable level for further processing. However, the viscosity of such PEO concentrates at ambient temperature is still more than 200 Pa·s, which is a fairly high value, close to the processing limit. 

**Figure 2:**
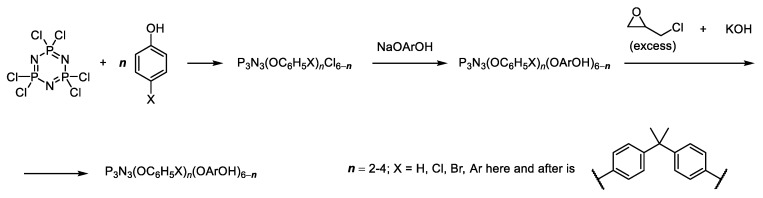
Replacement of the part of the chlorine atoms in chlorophosphazene with monophenol residues in order to increase the content of epoxycyclophosphazenes in a mixture with the organic epoxide [53].

The content of ECP in a mixture with the organic epoxide may be increased with simultaneous reducing of the content of residual chlorine, the functionality of HCP and its molecular weight, which was realized in our previous work [53] by replacing part of the chlorine atoms in chlorophosphazene with monophenol residues (Figure 2).

According to matrix-assisted laser desorption/ionization time-of-flight MALDI-TOF mass spectrometry, the main components of the oligomer formed are phosphazene-containing di-, tri- and tetraepoxides, the ratio of which in the reaction mixture can be varied by alternation of the value of *n*. The average content of epoxy groups in these PEO is 13%–14% by weight. Compositions based on diglycidyl ether of bisphenol A (DGEBA) and methyltetrahydrophthalic anhydride, containing 15% of the obtained oligomers in the epoxy component, compared to pure DGEBA, were characterized by more than 30% higher glass transition temperature and flexural strength, and at 75% content of PEO were non-combustible and had limiting oxygen index (LOI) of 30 [53]. However, the need to obtain and use of phenolates complicates the scalability of the described method.

Thus, the development of new scalable methods for obtaining epoxyphosphazene-containing epoxy resins based on available starting materials with reduced viscosity and improved technological properties is of great scientific and practical interest and may contribute to accelerating the widespread use of phosphazene-containing resins as components of high-tech flame-retardant polymer composite materials.

In order to exclude phenolate synthesis in the known method [53] (Figure 2) and to obtain pre-formulated PEOs with lower viscosity and greater phosphorus content in the present work, by analogy with [55,56], PEO was synthesized by direct interaction of HCP, phenol, bisphenol A, and epichlorohydrin.

## 2. Materials and Methods

**Figure 3:**
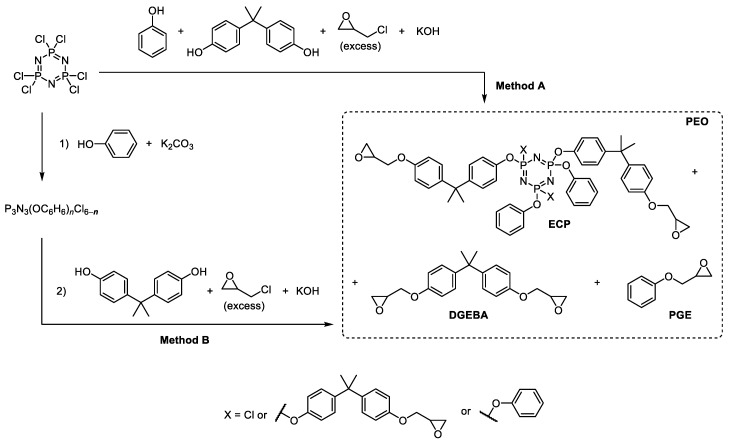
Single-stage synthesis of phosphazene-containing epoxy oligomers by direct interaction of hexachlorocyclotriphosphazene (HCP), phenol and bisphenol A in epichlorohydrin medium (this work).

In the present work PEO was synthesized by direct interaction of HCP, phenol and bisphenol A (BPA) in epichlorohydrin (ECH) medium as reagent and solvent in the presence of KOH with a role of both HCl acceptor and epoxy-forming reagent (Figure 3, Method A).

For comparison, stepwise replacement of chlorine atoms in HCP was carried out first on phenoxy groups, and then on the residues of bisphenol A in the presence of potassium carbonate as HCl acceptor on the first stage and with KOH on the second (Figure 3, Method B).

### 2.1. Starting Materials

Hexachlorocyclophosphazene—a white crystalline substance with m.p. of 113 °C; nuclear magnetic resonance (NMR) ^31^P-singlet spectrum with δ_P_ = 19.9 ppm, was obtained by the method [58]. Potassium hydroxide in the form of 90.0% pure white pellets (JSC KAUSTIK, Volgograd, Russia) was used without purification, the content of crystallization water determined by acid-base titration was about 10%. Epichlorohydrin (Solvay, Tavaux, France) with the content of the main substance of 99.8% was distilled before use as a colorless liquid, b.p. 118 °C. Potassium carbonate (Sigma-Aldrich, St. Louis, MI, USA) was dried in vacuum at 100 °C before use as a white crystalline substance in the form of powder, soluble in water. Bisphenol A (PJSC Kazanorgsintez, Kazan, Russia) was purified via repeated recrystallization from chlorobenzene to yield a product with m.p. of 156.5 °C. Phenol (Sigma-Aldrich, St. Louis, MI, USA) was distilled before use into white crystals, m.p. 40.0 °C. Solvents were purified according to known methods, their physical characteristics corresponded to the literature data [59].

### 2.2. Synthesis of Epoxyphosphazenes

#### 2.2.1. Single-Stage Synthesis of Phosphazene-Containing Epoxy Oligomers (Method A)

A 100 mL three-necked flask, equipped with a reflux condenser, a mechanical stirrer, and a thermometer, was charged with 1 g (0.0028 mol) of HCP, 2.68 g (0.0144 mol) of bisphenol A, 0.54 g (0.0058 mol) of phenol, and 65 mL of epichlorohydrin. The reaction mixture was heated to 50–55 °C and thermostated at this temperature for 30 min until all solids were completely dissolved. After that, 2.32 g (0.0414 mol) of potassium hydroxide was added and the process was conducted for 2 h at a temperature of 60 °C. At the end of the synthesis, the hot solution was filtered off and excess solvent was distilled off. The resulting mixture of epoxy oligomers was dried in vacuo at 85 °C. The reaction product is a slightly colored viscous liquid. The yield was 3.96 g (71%).

#### 2.2.2. Stepwise Synthesis of Phosphazene-Containing Epoxy Oligomers (Method B)

A 100 mL three-necked flask, equipped with a reflux condenser, a mechanical stirrer, and a thermometer, was charged with 1 g (0.0028 mol) of HCP, 0.54 g (0.0058 mol) of phenol, 0.95 g (0.0069 mol) potassium carbonate, and 65 mL of epichlorohydrin. The reaction mixture was heated to 60 °C and the process was conducted for 2 h at this temperature. The reaction mixture was then cooled to 50 °C, 3.28 g (0.0144 mol) of bisphenol A was charged and incubated at this temperature for 15 min until the bisphenol A was completely dissolved. After that, 2.32 g (0.0414 mol) of potassium hydroxide was added and the process was conducted for 3 h at a temperature of 60 °C. At the end of the synthesis, the hot solution was filtered off and excess solvent was distilled off. The resulting mixture of epoxy oligomers was dried in vacuo at 85 °C. The reaction product was a slightly colored viscous liquid. The yield was 4.18 g (75%).

### 2.3. Methods of Analysis

The ^31^P and ^1^H NMR spectra were measured in chloroform-d solutions with a Bruker AV-400 spectrometer (Bruker Corporation, Bremen, Germany) operating at 162 and 400 MHz, respectively. The signals due to the deuterated solvents were used as internal references. The chemical shifts of the signals were calculated relative to the signals of tetramethylsilane (^1^H) and phosphoric acid (^31^P), which were used as references. The spectra were processed with the help of the MestReNova Lab software package (Version 12.0.4, MESTRELAB RESEARCH, S.L, Santiago de Compostela, Spain).

MALDI-TOF mass spectrometric analysis was carried out on the Bruker Auto Flex II instrument (Bruker Corporation, Bremen, Germany).

The gas chromatography-mass-spectrometry (GC-MS) study was carried out on a VARIAN-3800 CP/4000 MS chromatograph-mass spectrometer (manufactured by Varian, Palo Alto, CA, USA). Separation of the substances was carried out using a VF-5ms chromatographic capillary column with a length of 30 m, an internal diameter of 0.25 mm, and a fixed-phase layer thickness of 0.25 μm. The carrier gas was helium. The injector temperature was 280 °C. The volume of the injected sample was 1 μL. The temperature of the column thermostat changed from 50 °C (1 min) to 280 °C (10 min) at a rate of 10 °C/min. The temperature of the ion source and the interface was 230 and 280 °C. The mass spectra of the substances were registered in the electron impact regime in the mass range 33–1000 Da. The processing of the obtained data was carried out with the usage of computer software “Varian MS Workstation”, the identification of the substances was carried out using the NIST 11 mass-spectra library.

The gel-permeation chromatography (GPC) was carried out on Shimadzu LC-20 Prominence (Kyoto, Japan) chromatograph equipped with refractometric and UV detector (a wavelength of 264 nm,) and a PSS column (SDV; 300 mm × 8 mm; 1000 A, separation within 100–60,000 Da), tetrahydrofuran (THF) was used as an eluent (1 mL/min). Molecular mass values were estimated with the use of a polystyrene calibration curve. Elemental analysis was carried out by spectrophotometry.

In the present paper, epoxy group content (in wt %) is the weight of oxirane groups (–CHCH_2_O) divided by the total molecular weight of epoxy resin. Thus, the theoretical content of epoxy groups was calculated by the formula:

(1) $$E_{calc}=\frac{43\times n}{M}100\%$$

where 43 and *M* are the molecular weights of the oxirane group and the whole epoxy resin molecule, respectively, and *n* is the number of oxirane groups in the epoxy resin molecule. The experimental epoxy group content was determined by the method of reverse acid-base titration [60,61,62]. In these methods, glycidyl groups are converted to chlorohydrin groups by dissolving the sample of epoxy resin in hydrochloric acid acetone solution followed by titration of the excess of hydrochloric acid with NaOH solution. The experimental epoxy group content is calculated by the formula:

(2) $$E_{exp}=\frac{\left(V-V_{0}\right)\times 43\times N}{g\times 1000}100\%$$

where *V* is the amount of NaOH solution for titration of a sample containing epoxy resin in mL; *V*_0_ is the amount of NaOH solution for titration of blank sample without epoxy resin in mL; 43 is the molecular weight of the oxirane group; *N* is the concentration of NaOH solution, mol⋅L^−1^; and *g* is the mass of epoxy resin sample in g.

The viscosity of epoxy oligomers was evaluated on a Reotest-2 rotational viscometer with a working cone-plane unit. The viscosity of the PEOs obtained in present and earlier papers was compared with conventional low molecular weight bisphenol A based epoxy resin D.E.R.^TM^ 332 epoxy resin (Dow Chemical, Midland, TX, USA) with the epoxy group content of 24.6%–25.1% and epoxy equivalent of 171–175 which is referenced as DGEBA and with ERISYS® Resorcinol diglycidyl ether RDGE (CVC Thermoset Specialties, Moorestown, NJ, USA) with the epoxy group content of 34.4%–36.4% and epoxy equivalent of 118–125.

## 3. Results and Discussion

**Figure 4:**
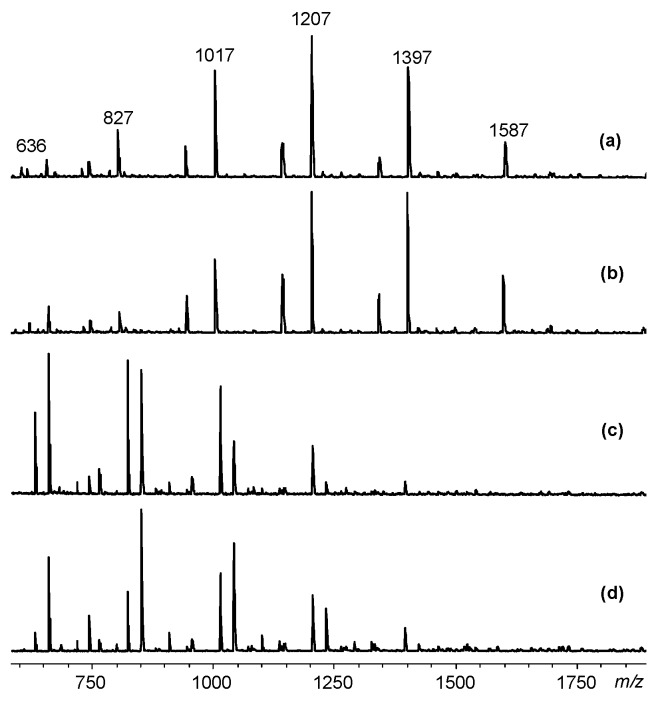
MALDI-TOF mass spectra of epoxycyclophosphazenes synthesized according to scheme A at molar ratios of HCP:phenol:bisphenol A = 1:2:5 (**a**), 1:2:6 (**b**), 1:4:3 (**c**), and 1:4:4 (**d**).

**Figure 5:**
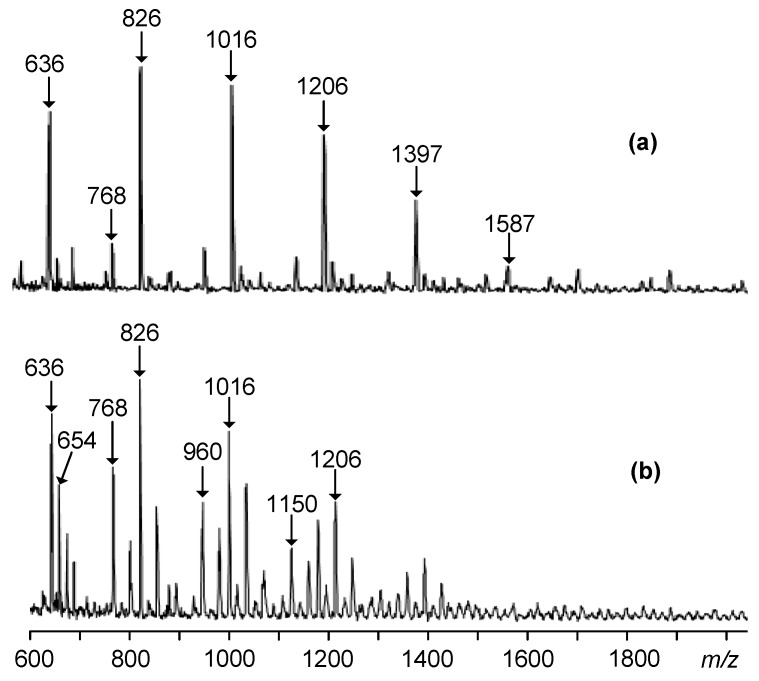
MALDI-TOF mass spectra of epoxycyclophosphazenes synthesized according to schemes A (**a**) and B (**b**) at molar ratios of HCP: phenol: bisphenol A = 1:3:5.

The composition and structure of the phosphazene component of the resulting mixture of epoxides were evaluated by combination of ^31^P NMR spectroscopy and MALDI-TOF spectrometry (Figure 4 and Figure 5) and the organic component by gas chromatography–mass spectrometry.

According to ^31^P NMR spectroscopy, irrespective of the initial mole ratio of HCP:phenol:bisphenol A compounds with pentasubstituted triphosphazene rings predominate in the phosphazene fraction (AB_2_ system with triplet δ_p_ = 20–26 ppm and doublet δ_p_ = 8–10 ppm) together with insignificant amounts of tetraaryloxy-substituted compounds (AB_2_ system with doublet at δ_p_ = 20–22 ppm and triplet at δ_p_ = 5–8 ppm).

Laser mass spectrometry data confirm the presence in the phosphazene fraction of mainly pentaaryloxy-substituted HCP, containing residues of phenol and diphenol in various proportions in the substituents near phosphorus atom (Figure 4, Table 1).

The content of compounds with different ratios of mono- and diphenolic aryloxy radicals as substituents near phosphorus atom can be controlled by varying the initial ratio of HCP:phenol:bisphenol A, as follows from Figure 4.

The quantitative composition of PEOs was determined from the relative intensity of the peaks on the MALDI-TOF spectra. On the example of the initial molar ratio of HCP:phenol:bisphenol A equal to 1:3:5 the content of individual compounds in the phosphazene fractions formed by methods A and B (Figure 3) is compared in Table 1.

More homogeneous is the composition of PEOs obtained according to method A (Figure 3): It contains four basic compounds with one, two, three, and four epoxy groups (Figure 5а and Table 1).

It is noteworthy that there are no compounds with four and five epoxy groups in the B synthesis product, but they appear in an amount up to 10 wt % of aryloxyphosphazenes with unsubstituted OH groups of bisphenol A radicals (peaks with *m/z* = 960 and 1150 in Figure 5b).

**Figure 6:**
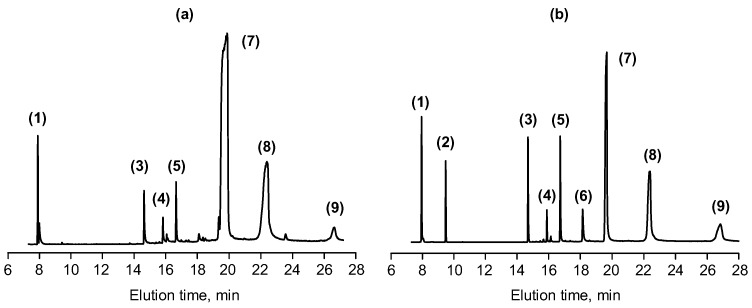
Chromatograms of organic epoxides obtained by schemes A (**a**) and B (**b**).

**Figure 7:**
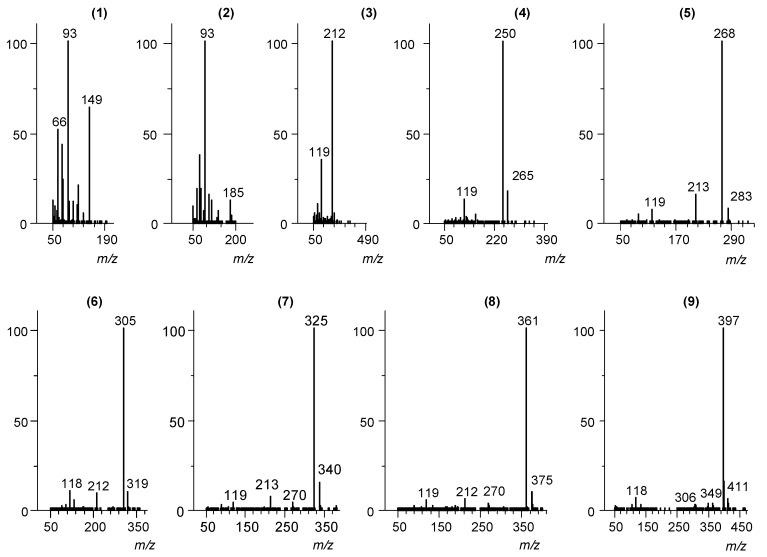
Mass spectra of fractions of organic epoxides synthesized according to methods A (Figure 3) and B (Figure 4). Spectrum numbers (1–9) correspond to the numbers of fractions on chromatograms, Figure 6.

Since the organic part of the synthesized epoxy-oligomers could not be evaluated well by MALDI-TOF, a gas chromatography-mass spectrometry method was used for its analysis (Figure 6 and Figure 7). As follows from Figure 6, the organic epoxide includes several compounds with different retention times in a chromatographic column.

There is insufficient information in the literature about the analysis of epoxy monomers by gas chromatography-mass spectrometry. However, given the fact that in order to achieve a satisfactory separation and to ensure the release of substances from the column, it was necessary to increase the temperature of the latter up to 280 °C, it can be assumed that fragmentation of the product components could occur not only as a result of electron impact during ionization, but also as a result of thermal exposure in a column.

The molecular weights of starting compounds and their derivatives formed as a result thermal exposure and electron impact estimated from the mass spectra allowed us to propose their chemical formulas which are listed in Table 2.

As follows from the data of gas chromatography-mass spectrometry, the main compounds in the organic part of the epoxide synthesized according to both schemes are mono- and diglycidyl ethers of bisphenol A with a predominant content of the latter (~70% and ~20% for methods A and B, respectively).

Phenylglycidyl ether (PGE) is present in a small amount only in the product synthesized according to method B (5%–10%). This indicates the preferential interaction of phenol not with epichlorohydrin, but with HCP at the initial stages of the process.

The results of the analysis of the electron impact mass spectra of the fractions (Figure 6) isolated from the chromatographic column allow us to draw the following conclusions (Table 2). The reaction products eluted from the column as a result of electron impact upon registration of the mass spectra may undergo some transformations, and the main among them is the transformation of the isopropylidene group into ethylidene:
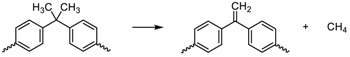


As a result, the majority of compounds fixed on mass spectra (peaks 3–8 in Figure 6) have a molecular weight of 16 units less than expected, which in our opinion is due to the elimination of CH_4_.

A similar decomposition of the isopropylidene group with the formation of a double bond was observed earlier under conditions of the synthesis bisphenol A based hydroxyaryloxycyclotriphosphazenes at 170 °C [60]:
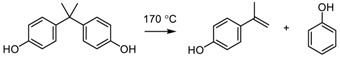


The main component of the organic fraction of the resulting epoxides is diglycidyl ether of bisphenol A (DGEBA), the highest yield (~70%) of which is achieved in the synthesis of method A with the simultaneous feeding of starting reagents.

The compounds’ formulas given in the sixth column of Table 2 show the elimination of methane from the corresponding compounds of the formulas given in the third column of this table.

It is noteworthy that in the reaction mixture there is practically no monoglycidyl ether of bisphenol A (MGEBA) with a molecular weight of 284, which corresponds to peaks with an insignificant intensity in the mass spectra of fraction 5. The main compound in fraction 5 for both synthetic schemes is the compound with a molecular weight of 268—monoglycidyl ether of 4,4′-dihydroxydiphenol-1,1-ethenyl.

As for compounds with one chlorohydrin group and the central ethenyl group (peak 8 on the chromatogram), the lack of a tendency to their dehydrochlorination is apparent due to both the heterogeneity of the process (KOH is insoluble in the reaction medium) and its insignificant duration (2 h).

From Figure 6 and Figure 7, it follows that the organic epoxide formed according to Method A (Figure 3) together with epoxycyclophosphazene is more pure—it contains about 90% mono- and diglycidyl ethers with minimal amounts of initial phenol and bisphenol A.

**Figure 8:**
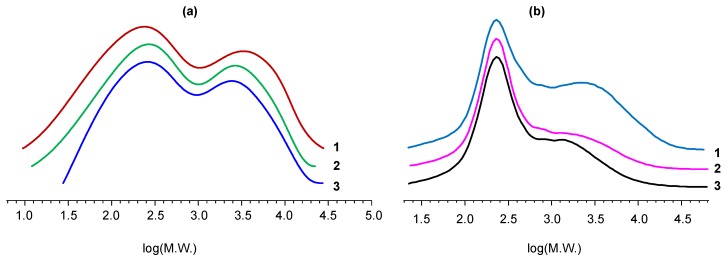
Gel-permeation chromatograms of PEO obtained in this by scheme A (**a**) with molar ratio of HCP:phenol:bisphenol A = 1:2:6 (1), 1:4:4 (2), 1:3:5 (3); and of resorcinol-based PEO (**b**) obtained in previous work [57] by interaction of HCP with resorcinol with molar ratio of HCP:resorcinol = 1:12 (1), 1:16 (2) and 1:24 (normalized to the height of the main peak).

The properties of different PEO obtained in this work and other PEOs [55,56,57] that can be synthesized using the one-pot method are summarized at Table 3. GPC was used (Figure 8) to determine the relative content of phosphazene and organic fractions (Figure 8a, Table 3). For comparison, the fractional composition of epoxyphosphazenes based on resorcinol obtained in a previous study [57] was also determined (Figure 8b, Table 3). 

The phosphazene component contents found from GPC data and calculated from the phosphorus content value are close to each other and are about 50%–60% for phenol-bisphenol A based product and are up to 45% for resorcinol-based product. The values of the weight average molecular weight according to GPC exceed the values obtained by the MALDI-TOF method, which can indicate both the limited applicability of the standard polystyrene calibration for these systems and the fact that high molecular weight particles may not be fixed by the MALDI-TOF method due to their limited volatility. However, the tendency of an increase in the average molecular weight with an increase in the content of the phosphazene component is confirmed by both GPC and MALDI-TOF, regardless of the nature of the starting reagents. It should be noted that the molecular weight values of the low molecular weight organic fraction determined by GPC and GC-MS are close and correspond to the theoretical assumption that its main component is diphenol diglydyl ether. From elemental analysis and GPC data it follows that with a comparable content of undesirable chlorine, the PEO obtained in this work is characterized by the both highest content of phosphorus (up to 5.4%) and phosphazene component (up to 61%).

The usage of phenol as additional reagent lead to reduced average fuctionality of the product of 1.9–2.2, which is closer to commercial DGEBA or RDGE resins when compared to 2.2–2.5 for the mixtures based on only bisphenol A or resorcinol. When compared to resorcinol-based phosphazene-containing epoxy oligomers, the values of viscosity of PEO obtained in this work are slightly higher. However, the viscosity of PEO obtained in this work at a ratio of HCP:phenol:bisphenol A = 1:4:4 and industrial bisphenol A based epoxy resin (such as diglycidyl ether of bisphenol A, DGEBA) are comparable (Table 3). At the same time, the viscosity of PEOs obtained at any ratio of HCP:phenol:bisphenol A is significantly lower than that of bisphenol A based phosphazene-containing epoxy resins. Thus, it can be expected that the processing properties of the PEO synthesized in this work will be as close as possible to ordinary bisphenol A based epoxy resins. As was shown in [53], such PEO are cured by conventional curing agents to form compositions with reduced flammability, increased glass transition temperature, flexural strength, and modulus while other characteristics of these compositions are at the level of commercially available epoxy materials.

## 4. Conclusions

The phosphazene-containing epoxy oligomers obtained in the present work have an epoxy group content within 15–16 wt %, phosphorus content within 4.6%–5.4% and epoxyphosphazene component content of 50%–60%. These phosphazene-containing epoxy oligomers may be cured by conventional curing agents to form materials with reduced flammability, while other characteristics of these compositions are at the level of commercially available epoxy materials [53]. The viscosity of obtained epoxyphosphazene-containing resins is comparable to conventional bisphenol A based epoxies, and is much lower in comparison to similar epoxyphosphazene resins based on bisphenol A. Thus, the obtained epoxyphosphazene resins may be used as a component of a binder for composite materials, adhesives, and paints.

## Figures and Tables

**Table 1:** The content of the phosphazene fractions of products synthesized by schemes A and B according to the data of MALDI-TOF-spectrometry. The molar ratio of HCP: phenol: bisphenol A = 1:3:5.

*m/z*	Compound Formula ^1^	Relative Content of the Compound (% weight) in the Product, Obtained via	
		Method A	Method B
636	P_3_N_3_Cl(OPh)_5_	12.3	3.6
654	P_3_N_3_Cl_4_(OPh)(OArOGly)	-	3.0
768	P_3_N_3_Cl_2_(OPh)_3_(OArOGly)	1.6	7.2
826	P_3_N_3_Cl(OPh)_4_(OArOGly)	14.0	8.6
958	P_3_N_3_Cl(OPh)_3_(OArOGly)(OArOGly′)	-	5.0
960	P_3_N_3_Cl(OPh)_3_(OArOH)(OArOGly)	-	8.5
1016	P_3_N_3_Cl(OPh)_3_(OArOGly)_2_	22.2	9.2
1150	P_3_N_3_Cl(OPh)_2_(OArOH)(OArOGly)_2_	-	3.0
1206	P_3_N_3_Cl(OPh)_2_(OArOGly)_3_	25.0	19.3
1397	P_3_N_3_Cl(OPh)(OArOGly)_4_	16.7	-
1587	P_3_N_3_Cl(OArOGly)_5_	1.0	-

^1^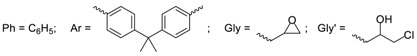.

**Table 2:** Results of chromatography-mass spectrometric analysis of organic fractions of reaction products obtained according to schemes A and B. Molar ratio of HCP:phenol:bisphenol A = 1:3:5.

Chromatography Data (Figure 6)					Mass-Spectrometric Data (Figure 7)			
Peak No	Elution Time (min)	Probable Formula ^1^ of the Compound at the Output of the Column	Calculated Molecular Weight	Content ^2^ of the Compound (wt%)	The Most Likely Formula	Calculated Molecular Weight	Observed *m*/*z* Values of the Products Obtained by Method	
							A	B
1	7.8	PhOH	93	2.0/7.6	PhOH	93	93	93
	7.9	PhOCH_2_CH=CH_2_	134	0.5/-	PhOCH_2_CH=CH_2_	134	133	none
2	9.3	PhOGly	150	0.9/4.8	PhOGly	150	none	149
3	14.6	HOArOH	228	1.0/6.3	HOAr′OH	212	212	212
4	15.8	HOArOGly	284	0.8/1.9	HOAr′OCH_2_CH=CH_2_	252	250	250
5	16.7	HOArOGly	284	2.0/8.2	HOAr′OGly	268	268 + 283	268 + 283
6	18.7	HOAr′OGly′	306.5	- /4.1	HOAr′OGly′	306.5	none	305
7	19.8	GlyOArOGly	340	66.8/45.9	GlyOArOGly	325	325 + 340	325 + 340
8	22.4	Gly′OAr′OGly	361	21.2/2.,1	Gly′OAr′OGly	361	361	361
9	26.8	Gly′OAr′OGly′	397	4.6/ -	Gly′OAr′OGly′	397	397	none

^1^ Ar’ = 
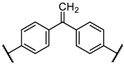
; ^2^ in the numerator for method A and in the denominator for method B.

**Table 3:** The properties of phosphazene-containing epoxy oligomers ^1^.

Raw Reagents Ratio	Mixture Average Functionality ^6^	Content (wt%)				Average Molecular Weight			Viscosity (Pa⋅s) at the Temperature of (°C)		
		Epoxy Group	P	Cl	Phosphazene Fraction ^2^	Entire Mixture ^3^ *M*_w_/*M*_n_	Organic Fraction	Phosphazene Fraction ^4^	20	40	70
DGEBA											
-	***2.0***	***24.6–25.1***	-	-	-	***346/-***	-	-	***5.83***	***0.86***	***0.06***
RDGE											
-	***2.0***	***34.4–36.4***	-	-	-	***243/-***	-	-	***1.10***	***0.11***	***0.03***
**HCP:BPA**	PEOs obtained by interaction of HCP with BPA and ECH [55,56,61]										
1:8	***2.5***	***17.1***	***3.1***	***2.7***	***49/49***	***1487/717***	***340*** ^5^	***1473***	-	***220***	***3***
1:12	***2.3***	***20.0***	***1.8***	***1.5***	***36/30***	***1212/681***		***1486***	-	***130***	***2***
1:16	***2.2***	***21.4***	***1.5***	***1.3***	***30/25***	***931/627***		***1492***	***440***	***78***	***2***
**HCP:Resorcinol**	PEOs obtained by interaction of HCP with resorcinol and ECH [57]										
1:12	***2.4***	***21.0***	***4.0***	***4.4***	45/***43***	2350/380	220 ^3^	***1054***	***8.33***	***6.15***	***0.36***
1:16	***2.3***	***28.6***	***3.0***	***2.4***	30/***32***	1260/260		***999***	***2.43***	***1.94***	***0.15***
1:24	***2.2***	***29.6***	***2.0***	***1.9***	23/***21***	1130/260		***957***	***1.71***	***0.45***	***0.05***
**HCP:PhOH:BPA**	PEOs obtained by interaction of HCP with BPA, phenol and ECH ^7^ (this work)										
1:2:6	2.2	16.1	4.6	2.2	61/54	4246/288	340 ^5^	1211	64.6	13.7	0.8
1:3:5	2.0	15.5	5.0	2.3	61/51	3459/413		1058	58.6	10.6	0.8
1:4:4	1.9	14.7	5.4	2.7	57/47	3248/317		930	9.4	6.0	0.8

^1^ This table in bold italics shows the literature data; ^2^ Found by gel-permeation chromatography (GPC) / Phosphorus content; ^3^ By GPC (See supplementary data for GPC curves); ^4^ By MALDI-TOF; ^5^ By GC-MS; ^6^ Calculated from phosphazene fraction content found from GPC, MALDI-TOF data, assuming that organic component’s functionality is 2; ^7^ By method A.
